# Genomic approaches to build de novo elite breeding gene pools from locally adapted landraces

**DOI:** 10.1007/s00122-025-05124-2

**Published:** 2026-01-07

**Authors:** Safiétou Tooli Fall, Alexander Kena, Brian R. Rice, Ghislain Kanfany, Cyril Diatta, Ndjido A. Kane, Allan K. Fritz, Geoffrey P. Morris

**Affiliations:** 1 Department of Soil and Crop Science, Colorado State University, Fort Collins, CO USA; 2 Centre d’Etudes Régional pour l’Amélioration de l’Adaptation à la Sécheresse (CERAAS), Institut Sénégalais de Recherches Agricoles, BP 3320, Thiès, Sénégal; 3 Crop and Soil Sciences, Kwame Nkrumah University of Science and Technology, Kumasi, Ashanti, PMB Ghana; 4 Department of Agronomy and Horticulture, University of Nebraska–Lincoln, Lincoln, NE USA; 5 Departement Productions Végétales et Agronomie, UFR des Sciences Agronomiques, de l’Aquaculture et des Technologies Alimentaires (S2ATA), Université Gaston Berger, Saint-Louis, Sénégal; 6 Institut Sénégalais de Recherches Agricoles (ISRA), Centre National de Recherches Agronomiques (CNRA) de Bambey, BP 53, Bambey, Sénégal; 7 Department of Agronomy, Kansas State University, Manhattan, KS USA

## Abstract

Many nascent breeding programs aim to achieve genetic gain by crossing locally-elite germplasm, but a lack of systematic approaches to develop elite gene pools from locally adapted varieties hinders their progress. Motivated by the observation of undesirable transgressive segregation in presumed elite crosses in Senegalese cereal breeding programs, we designed approaches for de novo development of elite gene pools from locally adapted landrace-derived germplasm. We first define two types of “elite” germplasm: *iso*-elite, phenotypically similar and genetically homogeneous for locally adapted traits (“attained traits”); versus *allo*-elite, phenotypically similar, but genetically heterogeneous for attained traits. Next, we defined two genomic approaches for de novo inference of elite gene pools: population-based genotypic inference (PGI) and QTL-based genotypic inference (QGI), and compared to a family-based phenotypic inference (FPI) approach. Using simulations that trace the evolution from locally adapted landraces to elite breeding lines, we evaluate the effectiveness of these strategies in nascent forward breeding programs. QGI accurately and cost-effectively identifies both *iso-* and *allo*-elite pairs, regardless of the underlying trait architecture, while PGI is less sensitive when trait architecture is oligogenic. Over ten cycles of phenotypic recurrent selection, programs based on *iso*-elite crosses consistently outperformed those based on *allo*-elite crosses for genetic gain. The findings highlight the value of trait genetic architecture knowledge for elite gene pool development and provide a practical roadmap for elite germplasm development in modernizing breeding programs.

**Supplementary Information:**

The online version contains supplementary material available at 10.1007/s00122-025-05124-2.

## Introduction

Intercrossing elite lines to develop improved cultivars is at the core of modern plant breeding (Rutkoski 2019). Despite widespread use of “elite” in plant breeding literature, however, there remains no universally accepted definition of “elite,” nor systematic guidance on how to develop elite germplasm (Acquaah 2012; Bernardo 2020; Falconer 1996; Falk 2010). The term is variously applied to denote superior phenotypic performance, the potential to produce superior progeny, and/or genetic similarity within breeding populations (or is not specifically defined) (Table S1). Advanced breeding programs, such as public-sector barley programs or private-sector corn breeding programs in North America and Europe, have typically managed narrow elite gene pools (Bornowski et al., 2021; Rasmusson and Phillips, 1997). By contrast, emerging breeding programs often begin with diverse landraces that are well-appreciated by local farmers and consumers; but the lack of clearly defined elite gene pools makes it difficult to design elite-by-elite crosses (Kane et al., 2022). Conversely, attempts to import “elite” germplasm from established breeding programs in different geographies have often failed because that germplasm is maladapted to local environments and cultural needs of smallholder farmers (Walker and Alwang 2015). Therefore, new approaches are needed to develop elite germplasm de novo from locally adapted landrace germplasm.

Genetic gain requires deliberate population improvement strategies that balance stabilizing selection to maintain favorable traits and directional selection to drive progress toward breeding goals (Falconer 1996; Hill 2010). Stabilizing selection helps preserve adaptive genetic variation by maintaining key allele frequencies, while directional selection promotes the accumulation of favorable alleles to improve target traits (Walsh and Lynch 2018a). The interplay between these selective forces underlies the genetic architecture of elite germplasm and influences long-term response to selection (Hill 2010; Walsh and Lynch 2018a). Breeding programs that manage this balance effectively sustain additive genetic variance, which according to the breeder’s equation, is critical for maximizing genetic gain (Lush 1943).

Landrace origins are shaped by a combination of farmer selection preferences, local environmental pressures, and seed exchange networks, which can promote genetic heterogeneity through diverse adaptation or lead to homogeneity under isolation or intensive selection (Nelimor et al. 2020; Rattunde et al. 2021). In crops such as sorghum, millet, and cowpea, this diversity is a critical resource, but it complicates the definition and development of elite germplasm. Phenotypic similarity among lines may mask underlying genetic heterogeneity (Brown 1989; Crossa et al. 2017), and individual-based selection risks overlooking this cryptic variation (R and B, 1981). In contrast, established programs have often used family-based selection to capture hidden structure and stabilize elite populations (Bernardo 2020; Comstock and Robinson 1948). These contrasting strategies highlight the need for emerging programs to adopt more deliberate frameworks that integrate diversity and structure in the development of elite gene pools.

Population genetic theory on parallel evolution is one promising area to inform frameworks for elite gene pool development. When distinct lineages from related founder populations are exposed to similar environmental pressures, they often evolve toward similar phenotypes, a common outcome in evolution known as parallel evolution (Wood et al. 2005). The evolutionary processes leading to genomic parallelism when multiple lineages independently evolve toward a similar phenotype in similar environments (Bohutínská et al. 2021). Individuals from different closely related founding populations adapt in similar ways under the same conditions with different genetics (Blount et al. 2018; Whiting et al. 2022). In addition, the pressure and direction of selection and genetic drift applied to individual quantitative trait loci (QTL) can give rise to alleles with an antagonistic effect for the trait, and thus increase the extent and frequency of transgressive segregation from crosses in domesticated populations (Dickinson et al. 2003). While parallel evolution has been studied in domestication and local adaptation, its implications for crop breeding remain largely underexplored.

Here, we present a framework for de novo elite gene pool development in emerging breeding programs through the use of molecular markers. Our objective was to identify accurate, efficient, and cost-effective strategies for inferring elite genetic relationships within diverse germplasm pools to optimize crossing decisions and accelerate genetic gain. To this end, we compared a phenotypic inference approach, family-based phenotypic inference (FPI), with two new genomic inference approaches, population-based genotypic inference (PGI) and QTL-based genotypic inference (QGI), evaluating their effectiveness across traits with differing genetic architectures. Our findings demonstrate that genotypic inference approaches can be successfully applied to establish elite gene pools, with particular strengths depending on the underlying genetic basis of target traits. These results highlight an opportunity to deploy genetic knowledge for de novo development of elite gene pools, offering practical guidance for emerging breeding programs aiming to balance eliteness with genetic diversity.

## Results

### Unexpected transgressive segregation from presumed elite breeding crosses

**Fig. 1:**
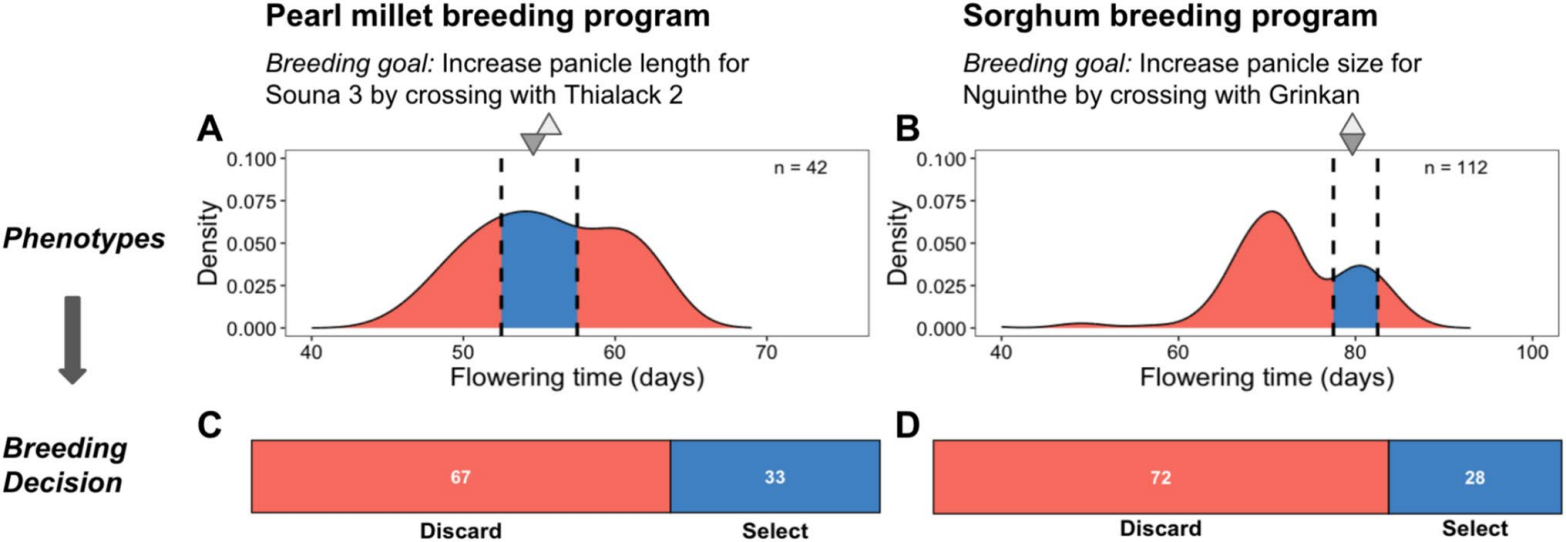
Undesirable transgressive segregation for an attained trait in progeny from presumed elite-by-elite crosses substantially reduces the number of candidates for selection. Phenotypic distribution of flowering time in progeny from presumed elite-by-elite crosses in millet (A; F_5_ progeny) and sorghum (B; F_7_ progeny) breeding programs in Senegal. For pearl millet, Souna 3 was crossed with Thialack 2 to improve panicle length while maintaining earliness and high yield. For sorghum, Nguinthe was crossed with Grinkan to improve panicle size while maintaining lateness. Flowering time is an attained trait in these programs, since the parents (triangles) are within the acceptable range of phenotypes (black dotted lines) according to the breeding product profiles. For both crops, substantial transgressive segregation was observed, with few progeny within the acceptable range (blue shading in A and B), and most progeny falling outside (red shading in A and B). Accordingly, the percentage progeny of discarded (red in C and D) versus selected (blue in C and D) progeny was high, with only 33 and 28% of the progeny for pearl millet and sorghum, respectively, selected as acceptable with respect to flowering time

To assess the status of eliteness within the germplasm of emerging breeding programs, we analyzed progenies from recent elite-by-elite crosses made by breeders, along with effectiveness of ongoing trait introgression efforts. The motivating example for this study was the observation of transgressive segregation in putative elite-by-elite Senegalese sorghum and pearl millet breeding programs (Fig. 1A–D). In pearl millet, a cross between the high-yielding, early-flowering variety Souna 3 and Thialack 2, valued for its long panicles, resulted in only 33% of F_5_ progenies flowering within the acceptable range (Fig. 1A), undermining expectations for combining favorable traits (Fig. 1C). A similar outcome occurred with a sorghum cross between two late-flowering lines, Nguinthe and Grinkan, intended to enhance panicle size (Fig. 1B and D). In both cases, the broad segregation for flowering time reduced the number of candidates for advancement, and therefore the effective population size (Fig. 1C–D).

### Conceptual model for genetic heterogeneity in elite germplasm: *iso-* versus *allo*-eliteness

**Fig. 2:**
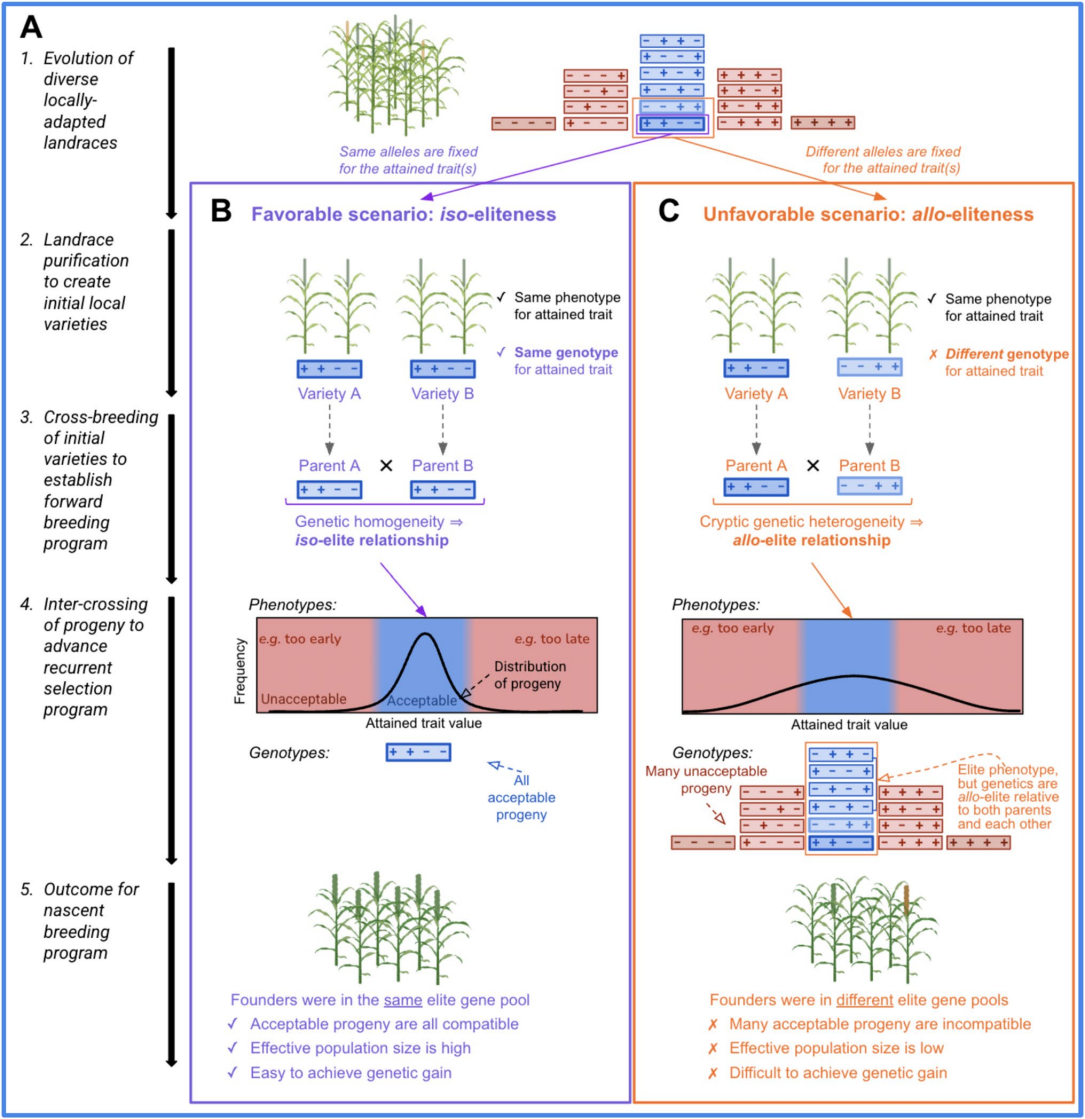
Evolutionary scenarios that lead to *iso-* versus *allo*-elite relationships in nascent breeding programs. **A** Schematic overview of the breeding trajectory, starting from landrace collection and parallel adaptation (Step 1), followed by landrace purification (Step 2), initial crossing of locally-elite lines (Step 3), intercrossing in recurrent breeding programs (Step 4), and culminating in the outcomes observed in nascent breeding programs (Step 5). Two distinct evolutionary scenarios are considered at each step: **B** *iso*-eliteness, characterized by genetic homogeneity among elite parents, and **C** *allo*-eliteness, characterized by cryptic genetic heterogeneity. Each rectangle represents the diploid genotype of a line, with “ + ” and “–” indicating favorable and unfavorable alleles, respectively, across four representative QTL for an attained trait. (Heterozygosity is ignored for simplicity). In the *iso*-elite scenario (**B**), genetically homogeneous elite parents produce progeny with a narrow phenotypic distribution, where most offspring fall within an acceptable range. By contrast, the *allo*-elite scenario involves genetically heterogeneous elite parents, resulting in a broader phenotypic distribution, with many progeny falling outside the acceptable range and being discarded

In many emerging breeding programs, particularly across sub-Saharan Africa early efforts over the past century focused on collecting and stabilizing locally adapted landraces into pure line varieties (Fig. 2A). These presumed elite lines were developed to consolidate key adaptive traits, such as flowering time, while enhancing agronomic performance traits like yield and disease resistance (Fig. 2A). Traits for which an acceptable phenotypic value has already been captured during this process are referred to here as “attained traits” (under stabilizing selection). By contrast, “desired traits” (under directional selection) are those for which breeding efforts aim to change the phenotypic value, often to meet farmer preferences or evolving environmental demands. Crosses are often made based on phenotypic similarity without accounting for underlying genetic relatedness or genotypic complementarity. We hypothesize that excessive transgressive segregation observed in crosses of similar parent lines may be explained by cryptic genetic heterogeneity shaped by parallel evolution in isolated breeding efforts (Fig. 2B–C).

Thus, we propose that two contrasting types of elite relationships may exist: *iso*- versus *allo*-elite. In the *iso*-elite cross scenario, repeated selection and genetic drift within locally adapted germplasm may result in elite lines that share similar genotypes at attained traits (Fig. 2B). Crosses between such genetically homogeneous lines typically produce narrow phenotypic distributions, with most progeny mostly resembling the parents in both trait phenotype and genetic makeup. This facilitates efficient selection and helps maintain a robust effective population size (Fig. 2B). By contrast, the *allo*-elite cross scenario would occur after a history of parallel adaptation of populations with the same phenotype, but different genotypes at attained traits. Here, elite lines derive from different ancestral lineages and carry distinct haplotypes, with alleles in repulsion phase (Fig. 2C). Crosses between such divergent lines can lead to extensive transgressive segregation due to the recombination of incompatible or novel allelic combinations (Fig. 2C). While this genetic novelty may occasionally yield superior individuals for desired traits, it more often results in progeny with unacceptable values for attained traits. This would lower the proportion of acceptable progeny, reducing effective population size, and therefore, genetic gain.

### Proposed approaches to infer *iso*- versus *allo*-elite relationships

**Fig. 3:**
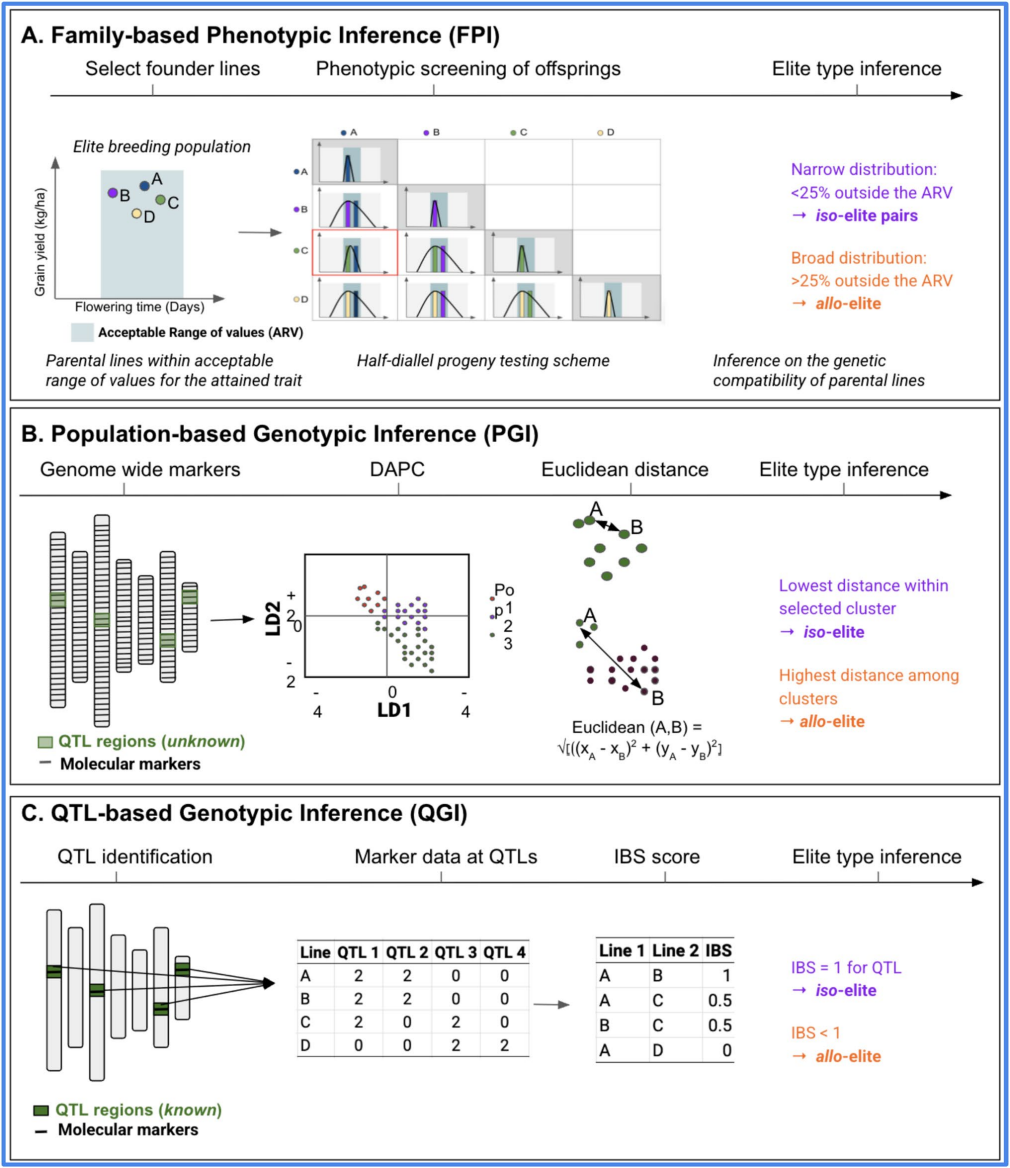
Schematic overview of approaches for inference *iso-* and *allo-*eliteness. **A** *Family-based phenotypic inference:* Elite lines were crossed using a half-diallel mating design. The *iso*- or *allo*-elite relationships were inferred from the phenotypic distribution of F_2_ progeny, and the accuracy of these inferences was evaluated using F_5_ progeny. Narrow phenotypic distributions were indicative of *iso*-eliteness, while broader distributions suggested *allo*-eliteness. **B** *Population-based genotypic inference *(PGI)*.* Whole-genome marker data were used to perform discriminant analysis of principal components (DAPC) and to calculate pairwise Euclidean genetic distances. Lineages from the same subpopulation, showing low genetic distances, were inferred to have *iso*-elite relationships, while those from different subpopulations, showing higher distances, were inferred to have *allo*-elite relationships. **C** *QTL-based genotypic inference *(QGI)*.* Identity-by-state (IBS) scores were calculated using markers located within known QTL. A score of IBS = 1 was interpreted as evidence of *iso*-eliteness, while IBS < 1 was interpreted as *allo*-eliteness

We propose three approaches to infer elite line relationships, each grounded in a distinct genetic rationale and suited to varying resource capacities:

- *Family-based Phenotypic Inference*: FPI would involve crossing elite lines in a diallel or partial diallel design and phenotypically evaluating the resulting segregating progeny (e.g., F_2_ to F_5_ generations) (Fig. 3A). The theoretical expectation is that *iso*-elite pairs, which share similar haplotypes, will produce narrow phenotypic distributions, while *allo*-elite pairs will yield broader segregation due to greater allelic divergence. Since this approach requires only classical breeding techniques (crossing, phenotyping, and selection), it may have been how (intentionally or unintentionally) classical breeders in the twentieth century developed elite gene pools. This approach provides inferences without genomic or molecular tools but is time-consuming and resource-intensive.
- *Population-based Genotypic Inference:* PGI would leverage genome-wide marker data to infer population structure among elite lines (Fig. 3B). We applied discriminant analysis of principal components (DAPC), a multivariate method that clusters individuals based on genetic variation without assuming Hardy–Weinberg equilibrium. DAPC maximizes between group variation while minimizing within group variance, allowing the identification of genetically homogeneous clusters. The rationale is that elite lines belonging to the same genetic cluster (i.e., same subpopulation) are more likely to share ancestral haplotypes, especially in genomic regions under selection. Such lines are thus inferred as having an *iso*-elite relationship, reducing the likelihood of unexpected segregation when crossed.
- *QTL-based Genotypic Inference:* QGI would use genetic similarity within known QTL that underlie attained traits that are essential for acceptability (e.g., flowering time, plant height, or grain color in cereals) (Fig. 3C). Identity-by-state (IBS) matrices are used to compare elite lines based on shared allelic states at locus-specific SNPs within trait-associated QTLs. This approach provides a more precise assessment of genetic compatibility by directly examining variation at underlying loci rather than genome-wide background. Elite lines that share high IBS values at relevant QTLs are considered *iso*-elite pairs with respect to the studied trait, whereas those with divergent alleles enabled allelic recombination increase the risk for transgressive segregation and therefore inferred *allo*-elite pairs. QGI is theoretically the most targeted and informative approach, but it depends on prior knowledge of QTL or quantitative trait nucleotides (QTNs) and access to high-quality genotypic data.

**Fig. 4:**
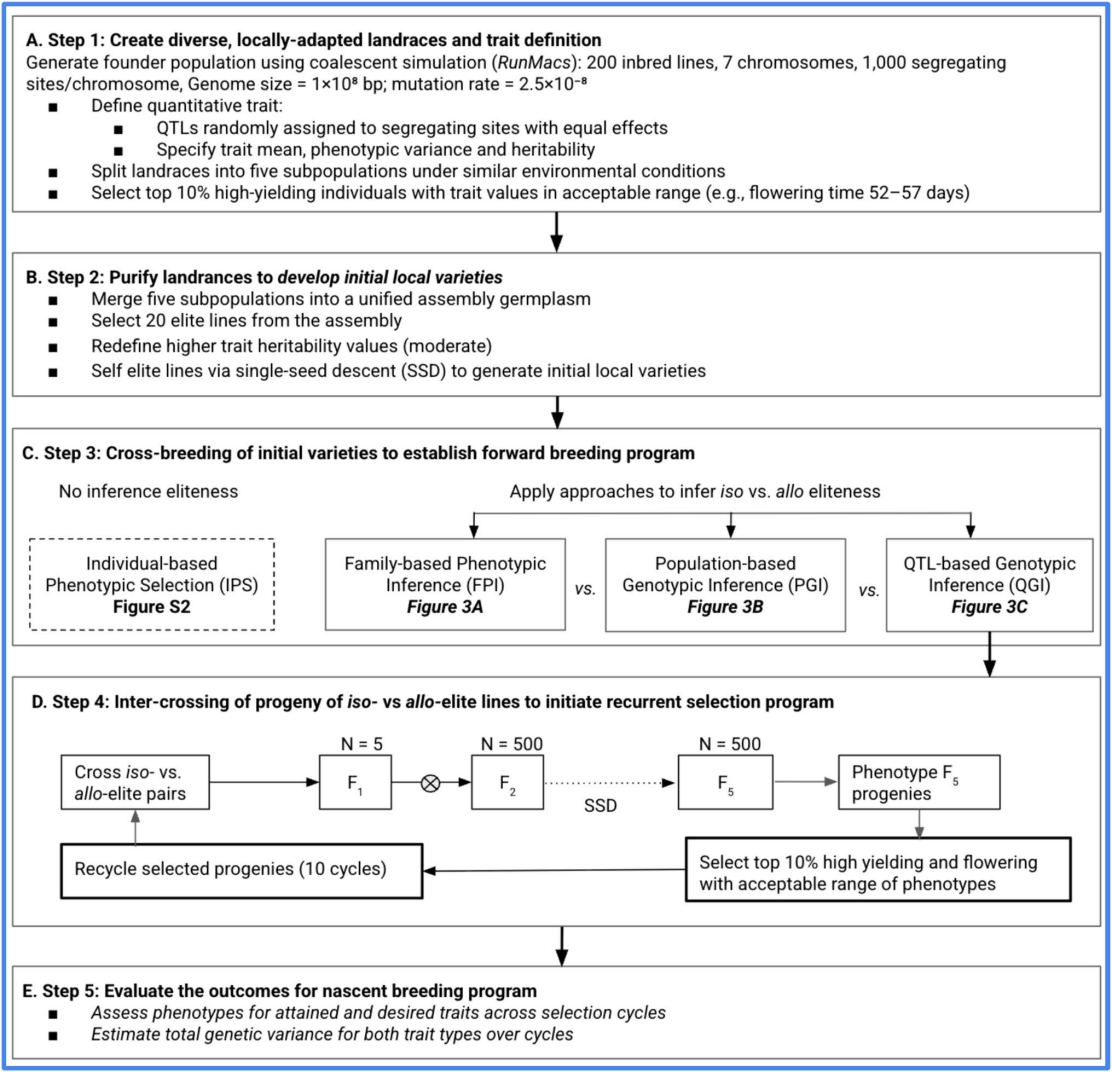
Simulation workflow for de novo creation, deployment, and evaluation of elite gene pools in nascent breeding programs. We conducted a five-step simulation process: **A** generation of diverse, locally adapted landraces with defined trait architecture; **B** purification and development of initial local varieties; **C** cross-breeding to initiate a forward breeding program; **D** intercrossing progeny with *iso*- or *allo*-elite relationships to launch phenotypic recurrent selection; **E** evaluate the outcomes for nascent breeding program

**Fig. 5:**
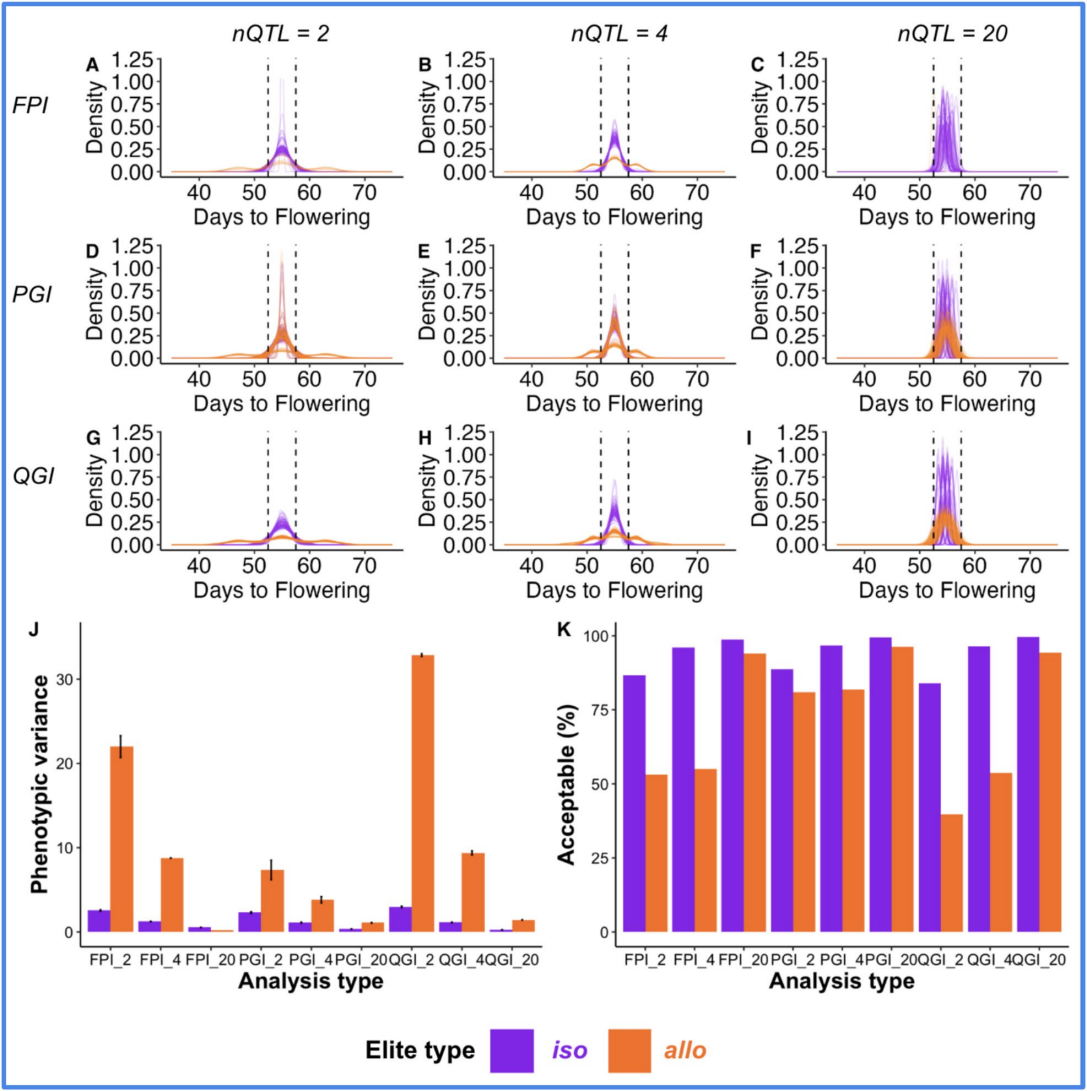
QTL-based genotypic inference (QGI) is the most accurate approach for distinguishing *iso-* and *allo*-elite relationships. Phenotypic distributions of F_5_ progenies were compared under three alternative inference approaches: family-based phenotypic inference (FPI) (panels **A**–**C**), QTL-based genotypic inference (QGI) (panels **D**–**F**), and population-based genotypic inference (PGI) (panels **G**–**I**). Each panel represents results under different genetic architectures defined by the number of QTLs: 2, 4, or 20. Purple lines represent the distribution of progeny from *iso*-elite crosses (expected to be narrow), while orange lines represent *allo*-elite crosses (expected to be broader). Average phenotypic variance (**J**) and acceptable percentage (**K**) for the simulated attained trait (flowering time) in F_5_ progeny was computed for each approach (FPI, PGI, QGI) and QTL (2, 4, 20) scenario, labeled as: FPI_2, FPI_4, FPI_20, PGI_2, PGI_4, PGI_20, and QGI_2, QGI_4, QGI_20. For each scenario, 500 individuals were phenotyped, and the phenotypic variance was averaged across 100 simulation replicates

To evaluate the potential effectiveness of these approaches, we simulated the steps in the evolution of nascent breeding programs (Fig. 2) and applied each of the approaches (Fig. 3) to the simulated breeding programs (Fig. 4; see Materials and Methods for details). These steps included: (1) creating diverse, locally adapted landraces and defining the trait (Fig. 4A), (2) purifying landraces to develop initial local varieties (Fig. 4B), (3) cross-breeding initial varieties to establish a forward breeding program (Fig. 4C), (4) intercrossing progeny of *iso*- versus *allo*-elite relationships to initiate a recurrent selection program (Fig. 4D), and (5) evaluating outcomes for the nascent breeding program (Fig. 4E).

### Family-based phenotypic inference is simple but limited to simple attained trait architecture

We evaluated the performance of family-based phenotypic inference (FPI) in distinguishing *iso-elite* and *allo-elite* relationships using F_2_ progeny distributions under simulated genetic architectures of 2, 4, and 20 QTLs (Fig. 5A–C). Phenotypic variance increased with the number of segregating loci, yet no consistent phenotypic pattern reliably separated *iso-elite* from *allo-elite* crosses. For example, F_2_ variance reached 21.99 for *allo-elite* versus 2.54 for *iso-elite* under 2 QTLs, and 8.77 versus 1.24 under 4 QTLs, indicating broader segregation in *allo-elite* crosses but substantial overlap between elite types. To operationalize the approach, we applied a breeder-defined threshold: Crosses were classified as *allo-elite* if more than 75% of F_2_ individuals fell outside the predefined acceptable phenotypic range. Under this criterion, FPI correctly identified elite type in 70% of cases when 4 QTLs were involved, but performance declined as trait complexity increased. At 20 QTLs, variance between *iso-elite* and *allo-elite* families converged (0.53 vs. 0.20), and > 94% of progeny remained within the acceptable range. Thus, while FPI offers a straightforward framework for elite-type inference, its accuracy is contingent on both trait complexity and specific phenotypic thresholds, motivating the exploration of genomic-based inference approaches such as PGI for improved resolution.

### Population-based genotypic inference can be effective for polygenic attained trait architecture

Next, we determined the accuracy of using the PGI approach. PGI failed to accurately detect *allo*-elite relationships at a lower number of loci (Fig. 5D–E). For QTL numbers of two and four, F_5_ progeny from *allo*-elite crosses showed a narrow distribution for some runs (Fig. 5D for QTL = 2; Fig. 5E for QTL = 4). Many families of inferred *allo*-elite crosses have narrow distributions leading to erroneous conclusions and thus unpredictable distribution in subsequent generations. In contrast, putative *iso*-elite crosses gave the expected distribution across all 100 runs (Fig. 5D for QTL = 2; Fig. 5E for QTL = 4). The accuracy of inference for *allo*-elite relationships improved as the number of QTL underlying the attained trait increased (Fig. 5F). At twenty QTL for the attained trait, *allo*-elite combinations were detected as accurately as *iso*-elite pairs and the accuracy of PGI was similar to QGI (Fig. 5F).

### QTL-based genotypic infers elite type across a range of trait genetic architectures

To determine whether QGI in known QTLs can be used to accurately infer *iso-* versus *allo-*elite pairs, we analyzed the phenotypic distribution in F_5_ generations for three genetic architecture scenarios. The progeny of *iso*-and *allo-*elite pairs inferred using QGI follows this prediction (Fig. 5G–I). Across all three genetic architecture scenarios, *iso-*elite crosses produce offspring with a narrow distribution for the attained trait (flowering time in days from sowing to flowering), almost all in the acceptable range of phenotypes. In contrast, *allo*-elite crosses generated offspring with a broad phenotypic distribution for flowering time when this trait is considered to have oligogenic architecture (Fig. 5G for QTL = 2, Fig. 5H for QTL = 4, and Fig. 5I for QTL = 20). In fact, when fewer high-effect variants segregate, the many possible allelic combinations give rise to a wider range of phenotypes for the trait. Thus, most of the progeny from these *allo-*elite crosses are outside the acceptable range of phenotypes and the number of loci is fixed to twenty (Fig. 5I). When the attained trait has multiple small-effect variants (nQTL = 20), the phenotypic distribution is still broader compared to the progeny of the *iso-*elite crosses. However, 94% of the offspring remain within the acceptable range of phenotypes as small-effect allele deviates the recombinants less from the initial average (Fig. 5K).

### QTL-based genotypic inference provides highest accuracy for elite-type inference

To assess the accuracy of elite-type inference, we compared phenotypic variance within progeny from *iso*- and *allo*-elite crosses across the three approaches (FPI, PGI, and QGI) under simulated genetic architectures involving 2, 4, and 20 loci (Fig. 5J). A reliable inference approach is expected to yield low phenotypic variance among *iso*-elite progeny and high variance among *allo*-elite progeny. FPI produced the highest overall phenotypic variance across all scenarios, with limited distinction between *iso*- and *allo*-elite crosses (Fig. 5J). While *allo*-elite crosses showed higher variance than *iso*-elite crosses at 2 and 4 loci, the variance among *iso*-elite progeny was unexpectedly high. At 20 loci, variance in *iso*-elite crosses remained elevated, suggesting that FPI becomes increasingly unreliable as trait complexity increases. PGI performed better than FPI, revealing significant differences in variance between *iso*- and *allo*-elite crosses (*p*-values: 0.0015, 0.0131, and 0.0007 for 2, 4, and 20 loci, respectively) (Fig. 5J). However, the magnitude of variance separation was modest compared to QGI. QGI consistently outperformed the other approaches, showing highly significant differences in phenotypic variance between *iso*- and *allo*-elite progeny at all locus levels (*p* < 0.001) (Fig. 5J). It also demonstrated a clear trend: Variance decreased with increasing locus number for both cross types, but remained distinctly lower for *iso*-elite and higher for *allo*-elite crosses.

### QTL-based genotypic inference is the most cost-effective approach when trait loci are known

We compared the cost-effectiveness of two genotypic inference approaches PGI and QGI against the FPI approaches, assuming prior knowledge of trait-associated loci (Table S3). When the number of loci was low (≤ 4), QGI was the most cost-effective, with estimated costs of $75 and $85 per line for 2 and 4 loci, respectively, compared to $125 for PGI. However, QGI costs rose with increasing locus number and surpassed PGI when 20 loci were involved ($165 per line). In contrast, when trait loci were unknown and required discovery via genome-wide association studies (GWAS) or linkage mapping, QGI became significantly more expensive $843 and $670 per line, respectively, exceeding both PGI and FPI (Table S4). Under comparable assumptions, FPI, which relies on crossing and phenotyping, incurred an estimated cost of $904 per line per environment (Table S3). Overall, PGI emerged as the most cost-effective approach when no prior knowledge of trait loci was available.

### Genetic gain under recurrent selection is more rapid for *iso*-elite crosses

To evaluate the impact of *iso*- versus *allo*-elite crossing strategies on genetic gain for a desired trait, we simulated 10 cycles of phenotypic recurrent selection, evaluating the performance of F_5_ progenies. We initially hypothesized that *iso*-elite crosses, despite potentially compromising genetic diversity for the attained trait, would still match or exceed genetic gain in the desired trait (e.g., yield potential). However, the relative performance of elite gene pool crossing strategy depended strongly on the subpopulation origin of the parental lines.

**Fig. 6:**
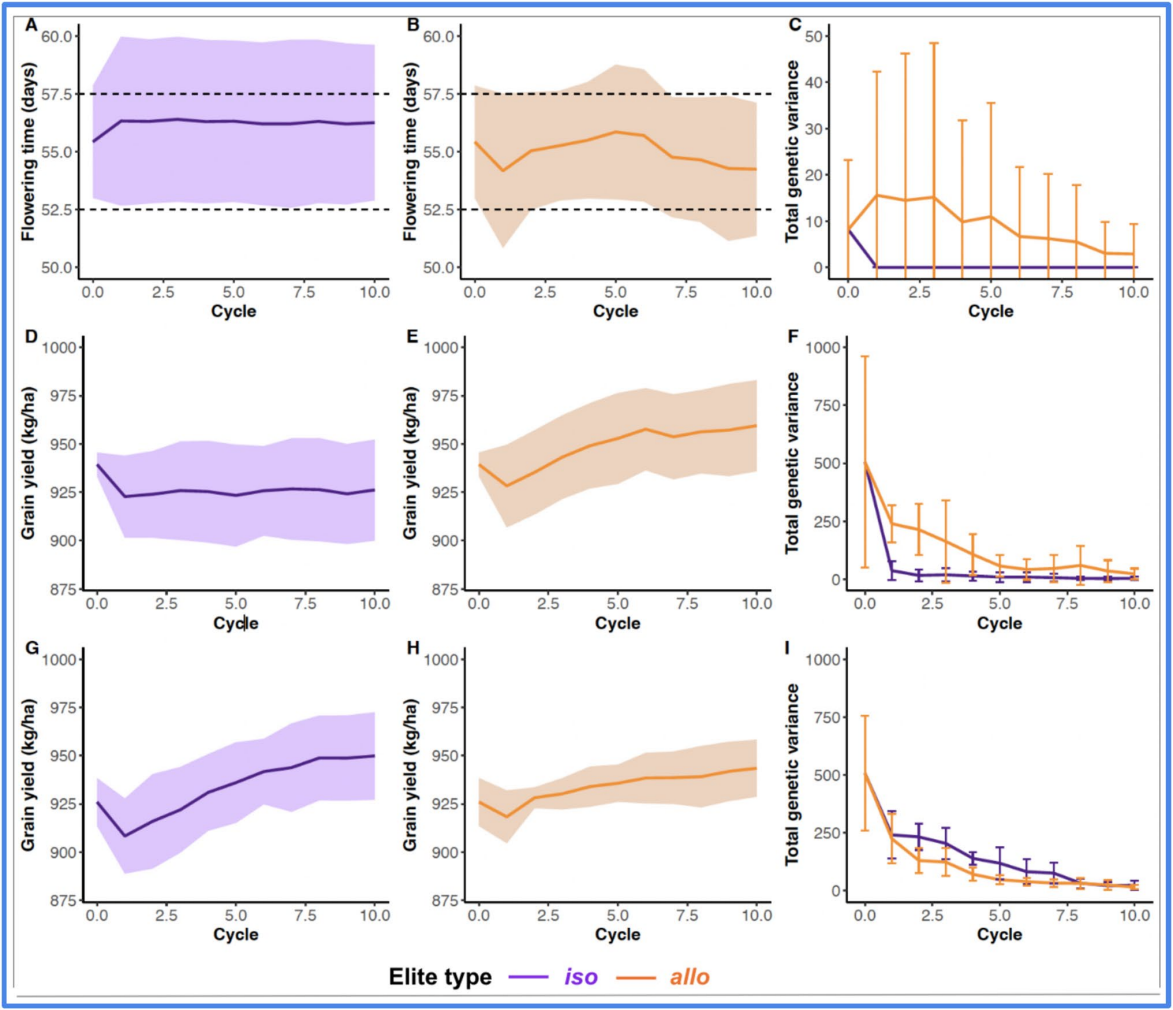
Genetic gain from *iso*-elite crosses surpasses *allo*-elite, but only when parents originate from different subpopulations. Average population mean and total genetic variance were tracked across selection cycles for progeny derived from *iso*- and *allo*-elite crosses. Panels **A**–**C**: Results for an oligogenic attained trait, **A** population mean from *iso*-elite crosses, **B** population mean from *allo*-elite crosses, and **C** total genetic variance over cycles (*iso* vs. *allo*). Panels **D**–**F**: Results for a polygenic desired trait and *iso*-elite parents from the same subpopulation, **D** population mean from *iso*-elite crosses, **E** population mean from *allo*-elite crosses, **F** total genetic variance over cycles (*iso* vs. *allo*). Panels **G**–**I**: Results for a polygenic desired trait and *iso*-elite parents from the different subpopulations, **G** population mean from *iso*-elite crosses, **H** population mean from *allo*-elite crosses, and **I** total genetic variance over cycles (*iso* vs. *allo*)

For the attained trait, progenies from *iso*-elite crosses maintained a stable population mean and narrow phenotypic variance across all cycles, indicating strong trait stabilization (Fig. 6A). The *allo*-elite progeny exhibited broader variation early on but gradually stabilized (Fig. 6B), while total genetic variance declined over time for both strategies, with *allo*-elite crosses showing an initial peak due to recombination (Fig. 6C). For the desired trait, outcomes differed based on whether *iso*-elite parents originated from the same or different subpopulations. When *iso*-elite parents shared the same subpopulation, *allo*-elite crosses yielded greater genetic gain over time (Fig. 6D–F). Although *allo*-elite progenies started with broader phenotypic variance (Fig. 6E), this variance declined over cycles and contributed to a stronger selection response. Genetic variance for the desired trait was higher in *allo*-elite crosses during the early cycles, supporting this advantage (Fig. 6F). However, when *iso*-elite parents originated from different subpopulations, they outperformed *allo*-elite crosses in final genetic gain (Fig. 6G–I). These crosses combined subpopulation diversity while retaining elite backgrounds, achieving both trait stability (Fig. 6G) and higher final population means (Fig. 6H), with comparable variance reduction over time (Fig. 6I).

## Discussion

### A modernized theoretical and applied framework for eliteness in breeding programs

Many nascent breeding programs, particularly in developing countries, are implementing individual-based phenotypic selection (IPS; Table 1, Fig. 3C) in crosses of locally adapted landrace-derived varieties, but seeing little success in terms of genetic gain or varietal adoption (Walker and Alwang 2015). Breeding efforts in low-income resource contexts have historically relied on phenotypic selection without access to genomic tools, so lines with locally-adaptive attained traits are often designated as *elite* based on phenotypic performance alone. Our findings show that crosses between such lines can produce unexpected segregation patterns, indicative of underlying genetic heterogeneity (Figs. 1, 2, 4). These outcomes suggest hidden genetic heterogeneity and contradict the notion that elite status equates to genetic homogeneity, a premise often overlooked or taken for granted in breeding literature (Falk 2010) (Table S1).

**Table 1:** Comparative analysis of breeding approaches based on key evaluation criteria

Approach	Infers eliteness	Uses genotypic information	Uses trait genetic information	Accurately infers *iso*-elite pairs	Accurately infers *allo*-elite pairs	Cost-effectively infers elite type
Individual-based phenotypic selection (IPS)	✗	✗	✗	NA	NA	NA
Family-based phenotypic inference (FPI)	✔	✗	✗	✗	✔	$$$
Population-based genotypic inference (PGI)	✔	✔	✗	✔	✗	$
QTL-based genotypic inference (QGI)	✔	✔	✔	✔	✔	$

Checkmarks (✓) indicate positive performance, while crosses (✗) indicate absence or limitation in each respective category, with relative cost indicated using dollar signs ($ = low, $$$ = high). NA = not applicable

To address this gap, we develop a framework for defining and operationalizing *eliteness* in nascent breeding populations. By explicitly separating these two categories of traits (attained versus desired), breeding strategies can be better aligned to harness recombination potential for desired traits while maintaining necessary levels of stability for attained traits (Figs. 2, 6). While we developed the theory and approaches with a focus on nascent programs in developing countries, similar genetic heterogeneity may exist within established networks of public programs, such as for winter wheat in the US Great Plains (Ayalew et al. 2020; Tessele et al. 2025), or even mature commercial hybrid maize programs (Technow et al. 2021).

Family selection remains a fundamental strategy in plant breeding, particularly in more mature programs where elite lines are extensively tested through their progeny performance (Acquaah, 2015). The theoretical basis of this approach assumes that phenotypic differences among families can be attributed to both genetic divergence and the compatibility of parental lines (Walsh and Lynch 2018a, b). The evaluation of FPI sought to clarify the extent to which these assumptions hold under varying genetic architectures, particularly in distinguishing between *iso*- and *allo*-elite relationships (Fig. 5). The rationale behind FPI aligns with classical family selection theory: that phenotypic distributions in progeny reflect underlying genetic structure, with narrower distributions expected from genetically similar (*iso*-elite) crosses, and broader segregation arising from genetically distinct (*allo*-elite) combinations (Falconer 1996; Walsh and Lynch 2018b).

To simulate the approach, we introduced a simple breeder-defined threshold: Crosses were classified as *allo-*elite if more than 75% of F_2_ individuals fell outside the predefined acceptable range of phenotypes. However, our results demonstrate that FPI effectively captured this expected pattern only under simple genetic architectures (2–4 QTLs), where *iso*-elite crosses showed narrow phenotypic variance and *allo*-elite crosses displayed broader segregation (Fig. 5). At higher genetic complexity (20 QTLs), this distinction largely disappeared, with nearly all simulated crosses behaving as *iso*-elite (only one *allo*-elite run out of 100). This confirms that while FPI performs as expected for oligogenic traits, its resolution declines sharply under polygenic control.

The current study was designed as an initial proof-of-concept to isolate conceptual contrasts among inference strategies (FPI, PGI, QGI) under a limited set of conditions (Fig. 4). Trait architectures were modeled as additive with equal-effect QTLs and without explicit genotyping error. Phenotypic error was implicitly captured through the evolving heritability parameter as genetic and environmental variances changed over cycles. While these simplifications provide an interpretable baseline for comparing inference methods, in practice genotyping error, phenotyping noise, and genotype × environment (G × E) interactions contribute substantially to inference uncertainty. Future work would need to extend the analyses to incorporate these sources of variation, testing the robustness of QGI and PGI under realistic breeding conditions (Clark et al. 2013; Swarup et al. 2021).

Our findings build on previous theoretical work emphasizing the interplay between within- and between-family genetic variance in shaping selection responses (Fehr, n.d.; Simmonds, 1996; Walsh and Lynch 2018a, b), and call attention to the need for more robust approaches that integrate genomic information with phenotypic evaluation to identify compatible genotypes. We used identity-by-state (IBS) as a strict operational definition of *iso-*elite and *allo-*elite: Two parents were considered *iso*-elite only if they shared all alleles across loci, ensuring that any difference in progeny variance reflected true allelic novelty rather than partial similarity. This simple IBS-based criterion provides a reproducible and interpretable baseline, while future work could incorporate genetic prediction and relationship matrices to refine elite-type inference.

Incorporating a breeder-defined cutoff (i.e., ≥ 25% of F_2_ individuals outside an acceptable range of phenotypes) improved FPI’s predictive utility to 70% accuracy in oligogenic scenario, suggesting a moderate practical value when trait phenotype is well characterized and thresholds are carefully chosen (Fig. 5). However, as the number of QTLs increased, predictive accuracy declined, highlighting the challenges of applying simple inference models to complex, polygenic traits. Importantly, the emergence of epistasis provides a mechanistic basis for these trends: As originally noted by Sewall Wright (Wright 1931), epistasis inevitably arises whenever selection acts toward an optimum. Thus, *iso*-elite genotypes can be viewed as occupying the same local epistatic peak, while *allo*-elite genotypes occupy different peaks within the adaptive landscape (Wade 1992; Wright 1932).

### Use genomics findings to guide de novo breeding elite gene pools development

The relevance of the QGI approach in this study is closely tied to the nature of the attained trait, which is controlled by a small number of major-effect loci. This aligns with longstanding debate in crop improvement regarding the value of trait mapping. While some have argued that mapping efforts offer limited utility for improving highly quantitative traits (Bernardo 2008; Rutkoski 2019), and that genome-wide selection may yield faster gains (Bernardo and Yu 2007), our findings suggest that when the architecture of an attained trait is oligogenic or monogenic, trait mapping is both relevant and impactful. Under such conditions, QGI targeting known loci offered high accuracy, biological relevance, and cost-effectiveness for elite-type inference (Fig. 5D–F, Table 1). These findings reinforce the value of causal gene identification in enhancing breeding efficiency, particularly in stress-resilience contexts where traits like flowering time or photoperiod sensitivity are key (Guitton et al. 2018).

Programs wishing to deploy QGI could leverage extensive trait mapping in orphan crops such as sorghum, pearl millet, and cowpea, which are targeted by many emerging breeding programs across sub-Saharan Africa and South Asia. In these crops, attained traits like flowering time, plant height, and drought tolerance have been successfully mapped due to rich phenotypic variation and segregation within traditional landraces (Boukar et al. 2016; Faye et al. 2021; Haussmann et al. 2002). Cloned loci such as *Ma1* (SbPRR37) and *Dw1* in sorghum (Murphy et al. 2011; Yamaguchi et al. 2016), and QTLs identified for flowering time and plant height in pearl millet (Varshney et al. 2017) and cowpea (Lo et al. 2018), could be used in QGI to define *iso*-elite gene pools. By integrating these mapped loci within QGI, programs can connect theoretical inference with real-world data resources such as the nested association mapping or introgression line resources (Ramstein et al. 2019) and the CGIAR Genomic Selection Platform. This approach grounds the framework in actionable resources, enabling breeders to design elite crosses based on measurable genetic relationships and trait-linked markers.

Our simulations of PGI provide qualified support for earlier proposals to use population structure to identify breeding gene pools from locally adapted landraces and global breeding germplasm (Faye et al. 2021). While this strategy is sound when polygenic trait alleles are already fixed (Fig. 2B), our results show that PGI performs poorly for oligogenic traits (Fig. 5D–E), where trait loci are few and may not align with principal genomic patterns. PGI’s reliance on overall genomic relationships makes it vulnerable when causal variation is confined to specific regions. Additionally, the accuracy of PGI was influenced by the clustering strategy DAPC performance varied based on the number of PC axes selected, a well-known issue that can lead to false subpopulation detection (Cullingham et al. 2023). Although we used the conservative *optim.a.score* method, this can overestimate dimensions under moderate-to-high gene flow. In scenarios where only a few QTLs underlie the trait, such false structure could obscure true elite-type relationships. Nevertheless, as QTLs represent a small fraction of genome-wide data used for clustering, the limitations of PGI under oligogenic control are consistent with theoretical expectations.

By contrast, QGI demonstrated superior performance by targeting genomic regions linked to specific trait loci. It was robust across trait architectures and retained accuracy even when PGI failed (Fig. 5G–I). Though PGI and QGI converged in performance for polygenic traits, QGI’s specificity made it a more reliable inference tool overall. Notably, QGI maintained high inference accuracy for monogenic and oligogenic attained traits, provided that trait loci had been mapped (Fig. 5D–F). This superior performance partly reflects a built-in advantage of the simulation design, where QGI was provided direct access to the causal loci underlying the attained trait. In real breeding applications, QGI would instead depend on markers linked to, rather than identical with, causal variants. The accuracy of such inference would therefore be affected by marker density, LD decay, and the presence of nonadditive effects such as dominance and epistasis (Liu et al. 2018; Ramstein et al. 2020; Viana and Garcia 2022). Benchmarking QGI with empirical datasets will thus be essential to assess how marker-based IBS estimates compare with true identity-by-descent (IBD) relationships and how this transition affects elite-type inference (Clark et al. 2013).

While our simulations incorporated a concave flowering-time fitness function to mimic adaptation to the local environments, this represents only a limited proxy for nonadditive effects. Real trait architectures frequently involve dominance, epistasis, and genotype-by-environment (G × E) interactions, which can alter the relationship between genotype and phenotype in complex ways. Extending the simulation framework to include these forms of nonadditivity will enhance realism and provide insight into how inference strategies perform when allelic effects are context-dependent or environment-specific. As the number of loci increases, inference precision can decline (Fig. 5J), due to the increased number of plausible haplotypes and variation in IBS scores across genome regions (Henden et al. 2018; Waples et al. 2019). Because IBS-based inference assumes that SNPs are in or near causal loci, the effectiveness of both QGI and future IBS-anchored approaches will depend strongly on accurate trait mapping and marker resolution (Clark et al. 2013). Future efforts integrating genomic prediction could relax this assumption, providing more flexible and probabilistic inference of elite relationships. In summary, our findings suggest that molecular marker tools can effectively guide elite gene pool development in emerging breeding programs, especially when the genetic architecture of key traits is well understood. QGI, in particular, stands out as a cost-effective and biologically grounded strategy for inferring *iso*- and *allo*-elite relationships especially when targeting a small number of high-priority traits. As breeding programs continue to face urgent demands with limited resources, approaches like QGI offer a practical path toward accelerating crop improvement.

Finally, our simulations demonstrate that the genetic diversity and the performance of *iso*-elite crosses depend strongly on the subpopulation origin of the parental lines. When *iso*-elite parents are drawn from the same subpopulation, they exhibit reduced genetic variability, which limits transgressive segregation for genetic yield potential and the likelihood of generating superior phenotypes for desired traits (Fig. 6D–F). This finding is consistent with prior findings that crossing closely related lines restricts segregational variance, leading to reduced long-term genetic gain particularly when the trait architecture is oligogenic and polygenic (Bernardo 2020; Clark et al. 2013). In contrast, *iso*-elite crosses between parents from different subpopulations retain the elite background while introducing meaningful genetic divergence, thereby increasing recombination opportunities and enhancing selection response (Fig. 6G–I). This suggests that such *iso*-elite crosses combine stability for attained traits with the genetic divergence typically targeted for desired traits. *Allo*-elite progenies, while initially more variable (Fig. 6E), also benefited from increased genetic variance early in selection, which enabled greater short-term gains in cases where *iso*-elite parents were genetically uniform. These findings emphasize that population structure within elite pools is a critical design consideration for maximizing genetic gain under recurrent selection (Falconer 1996; Swarup et al. 2021).

Beyond simulation, an important next step will be empirical validation of the proposed framework. Applying FPI, PGI, and QGI to structured germplasm resources such as multiple parents mapping populations (nested association mapping (NAM) populations or MAGIC) or diversity panels would provide a robust test of model performance under realistic conditions. These datasets combine known and unknown causal loci, varying marker densities, and imperfect phenotyping, thereby capturing multiple sources of uncertainty absent from our simulations. Such applications will clarify how inference accuracy degrades under genotyping error, incomplete tagging, or environmental noise, and will help calibrate the framework for use in practical breeding programs. In practice, breeding programs will also want to distinguish both *iso*-eliteness and *allo*-eliteness from what might be termed “*exo*-eliteness” germplasm that is truly elite for another geography or market class (e.g., with many broad-adaptation alleles and few deleterious variants) but not adapted to a given program's breeding targets.

## Conclusion

This study highlights that presumed elite lines in emerging breeding programs often harbor hidden genetic heterogeneity, leading to unexpected transgressive segregation when crossed. By distinguishing attained from desired traits, we demonstrate that elite-by-elite cross outcomes depend on parental genetic relationships. Among the inference methods evaluated, QTL-based genotypic inference (QGI) proved the most accurate and cost-efficient for identifying *iso-*elite and *allo*-elite relationships when trait loci are known. Simulations indicate that *iso*-elite crosses between parents from different subpopulations best balance stability in attained traits with recombination for desired traits, thereby optimizing long-term genetic gain. Grounded in real breeding program cost data, our analysis highlights how FPI, PGI, and QGI approaches can be operationalized through public genomic and germplasm resources. Together, these approaches provide a practical framework translating the conceptual distinction between *iso-* and *allo*-eliteness into a scalable strategy for guiding elite gene pool development in resource-limited programs.

## Materials and methods

### Simulating diverse locally adapted landraces

The MaCS software (Chen et al. 2009), implemented via the AlphaSimR package (Faux et al. 2016; Gaynor et al. 2021; Gorjanc et al. 2016), was used to generate an ancestral founding population of 500 outbred lines, setting the species history to GENERIC. This parameter enables the simulation of haplotype sequences shaped by historical changes in population size (Faux et al. 2016; Gaynor et al. 2021). Specifically, the effective population size was modeled to change over time: starting at 10,000 individuals 100,000 generations ago and decreasing stepwise to 6000, 1500, 500, and finally 100 individuals in the present, thus mimicking periods of reduced genetic diversity. The genome was modeled with 7 chromosomes, consistent with pearl millet (2*n* = 2*x* = 14 chromosomes), and each chromosome had 1000 random segregation sites. The physical and genetic lengths were set to 1 × 10^8^ base pairs and 1 Morgan, respectively. Haplotype sequences were generated using the Markovian Coalescent Simulator (MaCS), with a mutation rate of 2.5 × 10^−8^ per base pair (Chen et al. 2009). The founding population was simulated independently 100 times using the same parameters to mimic 100 independent breeding programs (Figs. 3 and 4).

For each simulation, two categories of traits were defined in the founding population: a moderately heritable attained trait (e.g., flowering time or plant height), and a lowly heritable desired trait (e.g., genetic yield potential). These traits were modeled based on phenotypic values observed in the Senegalese pearl millet local germplasm. The attained trait represented by flowering time was simulated with a mean of 55 days, variance of 10 days, and narrow-sense heritability (h2) of 0.6. The acceptable phenotypic range for flowering time was defined by setting minimum and maximum values at 52.5 and 57.5 days, respectively, to guide stabilizing selection. The desired trait grain yield was modeled with a mean of 900 kg/ha and a low heritability of 0.1, reflecting its complexity and high environmental influence in landraces. Grain yield potential for each individual was modeled based on its phenotypic value for the attained trait, with penalties applied to those whose trait values fell outside the acceptable range of phenotypes. The further an individual's phenotype deviates from the optimal mean and acceptable range for flowering time, the greater the penalty applied to its grain yield potential.

Three oligogenic genetic architecture scenarios were simulated for the attained trait, assigning 2, 4, or 20 quantitative trait loci (QTLs). In contrast, the desired trait was modeled as polygenic, controlled by 100 QTLs. All QTLs were independently and randomly distributed across the chromosomes. For each QTL scenario, equal additive effect sizes were simulated using an external function, defined by the following equations (see Figs. 3 and 4).

1 $${\mathrm{addeff}} = {\mathrm{rep}}\left( {{\mathrm{value,}}\;{\mathrm{total}}\_n{\mathrm{QTL}}} \right)$$

2 $${\mathrm{value}} = \left( {\left( {{\mathrm{max}}.t1 - {\mathrm{min}}.t1} \right) + 3} \right) / {\mathrm{total}}\_n{\mathrm{QTL}}$$

where total_*n*QTL is the number of loci controlling the trait, max.*t*1 and min.*t*1 represent the maximum and minimum values of the acceptable range of phenotypes. These equations ensure that the additive effects are scaled to maintain trait variability across different QTL numbers. Five subpopulations of 100 individuals each were derived from an ancestral founder population (Fig. 3). Within each subpopulation, individuals underwent random mating for 50 generations. At each generation, the top 10% of individuals based on grain yield and falling within an acceptable range for flowering time were selected and recycled for the next round of mating. This selection and mating scheme was applied independently within each subpopulation, mimicking local adaptation to similar environmental conditions while allowing genetic drift to shape allele frequencies over time.

### Simulating purification of landraces to create initial local varieties

Following 50 generations of selection and random mating within each of the five subpopulations, the resulting lines were merged to form a single assembly germplasm representing a broad base of locally adapted diversity. From this assembly germplasm, 200 individuals were selected to represent distinct landrace lines, capturing both phenotypic stability in the attained trait and variation in the desired trait. These selected lines formed the founding breeding population for subsequent improvement. To generate initial local varieties suitable for selection and further evaluation, each of the 200 lines was advanced through selfing using the single-seed descent (SSD) method, producing homozygous elite lines. This purification step served to stabilize trait expression and create a panel of fixed lines representing locally adapted landraces with elite potential.

### Simulating cross-breeding of initial varieties without eliteness inference

To simulate conventional improvement strategies without prior inference of elite relationships (individual-based phenotypic selection; IPS), the top 10% of highest-yielding lines among 200 purified lines were designated as elite. Trait donor lines were selected from within this elite group, each carrying beneficial alleles for specific agronomic traits of interest (e.g., drought tolerance, disease resistance). A forward breeding approach was employed: Selected local varieties were crossed with these trait donors, and the resulting progeny underwent phenotypic screening to identify individuals demonstrating superior performance for both introduced and target traits. Progeny exhibiting favorable phenotypes were advanced through standard breeding pipelines, with top-performing individuals selected as candidates for multilocation and advanced field trials. This strategy reflects typical breeding practices in the absence of prior elite-type inference and served as a comparative baseline for evaluating the effectiveness of structured gene pool approaches.

### Phenotypic inference of elite gene pools

FPI was deployed using a half-diallel mating design to evaluate all possible combinations among selected elite parental lines. The F_2_ progenies derived from each cross were assessed to characterize the distribution of phenotypic variation within families. These distributions were used to infer the relationships (*iso* vs. *allo*) and define gene pools. Crosses that produced F_2_ populations with narrow phenotypic distributions were inferred to involve parents in a *iso* relationships, while those with broader distributions were interpreted as *allo*-elite relationships, reflecting greater segregation variance. To validate these initial inferences, we examined the distribution of phenotypic variation within F_5_ progenies derived from the same crosses.

### Genotypic inference of elite gene pools

For QGI, we extracted the genotype information in the QTL and then calculated the identity-by-state (IBS) for each pair of individuals for each marker position using the cluster function daisy() from the R package (Fig. 3). Pairs of individuals with the highest IBS indices equal to 1 were considered *iso-*elite pairs (Fig. 3). In contrast, pairs of individuals with the IBS indices inferior to 1 were considered *allo-*elite pairs (Fig. 3).

For PGI, a data matrix of molecular markers across all segregating sites was used to first identify clusters using successive K-means with the function find.clusters(). A first discriminant analysis of principal components (DAPC) was performed with these clusters using the dapc() function and then used optim.a.score() to define the optimal number of principal components (PCs). A second DAPC was performed using the defined optimal number of PCs. Euclidean distance was used to infer *iso*- and *allo*-elite relationships based on clustering (Fig. 3). The Euclidean distance between individuals between and within clusters using the following equation:

$${\text{Euclidean }}\left( {A, \, B} \right) \, = \, \surd [(x_{A} - \, x_{B} )^{2} + \, \left( {y_{A} - \, y_{B} } \right)^{2} ]$$

The pairs of individuals with the lowest and highest Euclidean distance within the cluster with the highest proportion of successful reassignment were inferred as having *iso-*elite and *allo-*elite relationships, respectively (Fig. 3).

### Analysis of the phenotypic distribution of offspring

Five pairs of putative *iso-* and *allo-*elite combinations were crossed, respectively, to sequentially generate one F_1_ progeny and selfed to obtain 100 F_2_ progeny then, 100 F_5_ individuals per single-seed descent from each pair (Figs. 3 and 4). The phenotypic distribution for flowering time of F_5_ progeny of *iso-* and *allo-*elite crosses was analyzed using a histogram in *ggplot2* R package, respectively. The accuracy of elite-type prediction was therefore assessed based on the phenotypic distribution. A narrow distribution was expected for F_5_ progeny derived from *iso*-elite crosses, whereas a broad distribution was expected for those derived from *allo*-elite crosses (Fig. 1C–D).

### Comparing costs of de *novo* gene pool strategies

We estimated the costs of defining breeding gene pools for 50 elite lines using QGI and PGI and compared them to FPI. Cost estimates were based on the millet breeding program budget in Senegal during the 2016/2017 season (Table S2). Figure 4 outlines the sequence of steps considered for each strategy. The phenotypic approach involved crossing the 50 elite lines with four required control lines (depending on the trait) using a half-diallel mating design. F_1_ seeds were harvested from each cross and then advanced to F_5_ generation progenies, following the procedures previously described (Fig. 4). The phenotypic distribution of the F_5_ progenies was then analyzed for the trait of interest.

For the genotypic approaches, we considered two scenarios: (i) major QTLs already known, and (ii) QTLs unknown and requiring discovery. The latter scenario includes additional steps such as forward genetic methods GWAS and biparental QTL mapping as well as reverse genetic approaches like allele mining and comparative genomics supported by functional genomics. In this study, cost estimates were based solely on forward genetics. For GWAS, we estimated the cost of genotyping and phenotyping a diversity panel of 300 lines across two sites over two years. For biparental QTL mapping, we simulated the development and evaluation of 250 F_2_ individuals, including genome-wide genotyping and trait phenotyping. Once major QTLs were identified, the next steps included genotyping the 50 elite lines at these specific regions for allele screening and computing similarity matrices for QGI analysis. To estimate PGI costs, we assumed genome-wide genotyping of the 50 elite lines followed by PGI analysis for elite clustering.

Genotyping costs were estimated at $20 per line for genome-wide data and $5 per line for each QTL-specific assay based on previous genotyping activities conducted in Senegalese the sorghum breeding program. Phenotyping costs were estimated at $64 per line, based on the same 2016/2017 Senegalese pearl millet program budget. The experimental design used to derive phenotypic cost estimates was an alpha-lattice design (7 × 13), with three replications. Each elementary plot consisted of two rows of 8 mounds, flanked by two border rows, with 90 cm spacing between rows and between mounds. Costs included all field operations in a rainfed environment from land preparation to harvest covering labor, inputs, and equipment (Table S2).

### Simulating intercrossing to initiate recurrent selection program

To initiate the recurrent selection program, three additional attained traits were simulated, resulting in a total of four attained traits and one desired trait modeled in the ancestral founding population. The purification process to create initial local varieties was conducted as previously described. QGI was used exclusively to infer *iso-* and *allo-*elite relationships among parental lines. These inferred elite parent pairs were then used to initiate a phenotypic recurrent selection (PRS) program. In cycle 0 (C0), two scenarios were simulated independently: one starting with five *iso-*elite pairs and the other with five *allo-*elite pairs. From each cross, one F_1_ plant was generated and selfed to produce 100 F_2_ individuals via single-seed descent, resulting in a total of 500 F_5_ individuals per scenario (Fig. 4). These F_5_ individuals were advanced through subsequent generations via the same method. At each cycle, selection was based on performance for the desired trait (e.g., grain yield), while maintaining attained traits within an acceptable range of phenotypes. This applied directional selection on the desired trait and stabilizing selection on the attained traits. The phenotypic traits were modeled as independent traits in the simulation. A total of ten selection cycles were simulated for each scenario to evaluate the long-term effectiveness of the phenotypic recurrent selection strategy. The outcomes of the nascent breeding program were evaluated by tracking phenotypic trends for both attained and desired traits across selection cycles using *ggplot2* in R. In addition, total genetic variance for these traits was extracted and visualized to assess the impact of selection on genetic diversity over time.

## Supplementary Information

Below is the link to the electronic supplementary material.

Supplementary file1 (DOCX 18 KB)

## References

[CR1] Acquaah G (2012) Principles of plant genetics and breeding, 2nd edn. Wiley-Blackwell, Hoboken

[CR52] Acquaah G (2012) Principles of Plant Genetics and Breeding, 2, edition. Wiley-Blackwell, Hoboken, NJ

[CR2] Ayalew H, Sorrells ME, Carver BF, Baenziger PS, Ma X-F (2020) Selection signatures across seven decades of hard winter wheat breeding in the Great Plains of the United States. Plant Genome 13(3):e20032. 10.1002/tpg2.20032 33217215 10.1002/tpg2.20032 PMC12807241

[CR3] Bernardo R (2008) Molecular markers and selection for complex traits in plants: learning from the last 20 years. Crop Sci 48(5):1649–1664. 10.2135/cropsci2008.03.0131

[CR4] Bernardo R (2020) Breeding for quantitative traits in plants, 3rd edn. Stemma Press, New York

[CR5] Bernardo R, Yu J (2007) Prospects for genomewide selection for quantitative traits in maize. Crop Sci 47(3):1082–1090. 10.2135/cropsci2006.11.0690

[CR6] Blount ZD, Lenski RE, Losos JB (2018) Contingency and determinism in evolution: replaying life’s tape. Science 362(6415):eaam5979. 10.1126/science.aam5979 30409860 10.1126/science.aam5979

[CR7] Bohutínská M, Vlček J, Yair S, Laenen B, Konečná V, Fracassetti M, Slotte T, Kolář F (2021) Genomic basis of parallel adaptation varies with divergence in *Arabidopsis* and its relatives. Proc Natl Acad Sci USA 118(21):e2022713118. 10.1073/pnas.2022713118 34001609 10.1073/pnas.2022713118 PMC8166048

[CR54] Bornowski N, Micel KJ, Hamilton JP, Ou S, Seetharam AS, Jenkins J, Grimwood J, Plott C, Shu S, Talag J, Kennedy M, Hundley H, Singan VR, Barry K, Daum C, Yoshinaga Y, Schmutz J, Hirsch CN, Hufford MB, de Leon N, Kaeppler SM, Buell CR (2021) Genomic variation within the maize stiff-stalk heterotic germplasm pool. The Plant Genome 14:e20114. 10.1002/tpg2.20114 34275202 10.1002/tpg2.20114 PMC12806955

[CR8] Boukar O, Fatokun CA, Huynh BL, Roberts PA, Close TJ (2016) Genomic tools in cowpea breeding programs: status and perspectives. Front Plant Sci. 10.3389/fpls.2016.00757 27375632 10.3389/fpls.2016.00757 PMC4891349

[CR9] Brown AHD (1989) Core collections: a practical approach to genetic resources management. Genome 31(2):818–824. 10.1139/g89-144

[CR10] Chen GK, Marjoram P, Wall JD (2009) Fast and flexible simulation of DNA sequence data. Genome Res 19(1):136–142. 10.1101/gr.083634.108 19029539 10.1101/gr.083634.108 PMC2612967

[CR11] Clark SA, Kinghorn B, Van Der Werf JH (2013). Comparisons of identical by state and identical by descent relationship matrices derived from SNP markers in genomic evaluation. Association for the Advancement of Animal Breeding and Genetics (AAABG). https://rune.une.edu.au/web/handle/1959.11/14477

[CR12] Comstock RE, Robinson HF (1948) The components of genetic variance in populations of biparental progenies and their use in estimating the average degree of dominance. Biometrics 4(4):254–266. 10.2307/3001412 18108899

[CR13] Crossa J, Pérez-Rodríguez P, Cuevas J, Montesinos-López O, Jarquín D, Campos G, Burgueño J, González-Camacho JM, Pérez-Elizalde S, Beyene Y, Dreisigacker S, Singh R, Zhang X, Gowda M, Roorkiwal M, Rutkoski J, Varshney RK (2017) Genomic selection in plant breeding: methods, models, and perspectives. Trends Plant Sci 22(11):961–975. 10.1016/j.tplants.2017.08.011 28965742 10.1016/j.tplants.2017.08.011

[CR14] Cullingham C, Peery RM, Miller JM (2023) A roadmap to robust discriminant analysis of principal components. Mol Ecol Resour 23(3):519–522. 10.1111/1755-0998.13724 36282622 10.1111/1755-0998.13724

[CR15] Dickinson HG, Hiscock SJ, Crane PR, Rieseberg LH, Widmer A, Arntz AM, Burke JM (2003) The genetic architecture necessary for transgressive segregation is common in both natural and domesticated populations. Philos Trans R Soc Lond B Biol Sci 358(1434):1141–1147. 10.1098/rstb.2003.1283 12831480 10.1098/rstb.2003.1283 PMC1693210

[CR16] Falconer DS (1996) Introduction to quantitative genetics. Pearson Education, London

[CR17] Falk DE (2010) Generating and maintaining diversity at the elite level in crop breeding. Genome 53(11):982–991. 10.1139/G10-081 21076514 10.1139/G10-081

[CR18] Faux A-M, Gorjanc G, Gaynor RC, Battagin M, Edwards SM, Wilson DL, Hearne SJ, Gonen S, Hickey JM (2016) AlphaSim: software for breeding program simulation. Plant Genome. 10.3835/plantgenome2016.02.0013 27902803 10.3835/plantgenome2016.02.0013

[CR19] Faye J, Maina F, Akata E, Sine B, Diatta C, Mamadou A, Marla S, Bouchet S, Teme N, Rami J, Fonceka D, Cisse N, Morris G (2021) A genomics resource for genetics, physiology, and breeding of West African sorghum. Plant Genome. 10.1002/tpg2.20075 33818011 10.1002/tpg2.20075 PMC12807439

[CR20] Gaynor RC, Gorjanc G, Hickey JM (2021) AlphaSimR: an R package for breeding program simulations. G3 Genes|genomes|genetics 11(2):jkaa017. 10.1093/g3journal/jkaa017 33704430 10.1093/g3journal/jkaa017 PMC8022926

[CR21] Gorjanc G, Jenko J, Hearne SJ, Hickey JM (2016) Initiating maize pre-breeding programs using genomic selection to harness polygenic variation from landrace populations. BMC Genomics 17:30. 10.1186/s12864-015-2345-z 26732811 10.1186/s12864-015-2345-z PMC4702314

[CR22] Guitton B, Théra K, Tékété ML, Pot D, Kouressy M, Témé N, Rami J-F, Vaksmann M (2018) Integrating genetic analysis and crop modeling: a major QTL can finely adjust photoperiod-sensitive sorghum flowering. Field Crops Res 221:7–18. 10.1016/j.fcr.2018.02.007

[CR23] Haussmann BIG, Mahalakshmi V, Reddy BVS, Seetharama N, Hash CT, Geiger HH (2002) QTL mapping of stay-green in two sorghum recombinant inbred populations. Theor Appl Genet 106(1):133–142. 10.1007/s00122-002-1012-3 12582881 10.1007/s00122-002-1012-3

[CR24] Henden L, Lee S, Mueller I, Barry A, Bahlo M (2018) Identity-by-descent analyses for measuring population dynamics and selection in recombining pathogens. PLoS Genet 14(5):e1007279. 10.1371/journal.pgen.1007279 29791438 10.1371/journal.pgen.1007279 PMC5988311

[CR25] Hill WG (2010) Understanding and using quantitative genetic variation. Philos Trans R Soc Lond B Biol Sci 365(1537):73–85. 10.1098/rstb.2009.0203 20008387 10.1098/rstb.2009.0203 PMC2842708

[CR55] Kane Ed N, Daniel Foncéka E, Timothy Dalton E (2022) Crop Adaptation and Improvement for Drought-Prone Environments. NPP eBooks. https://newprairiepress.org/ebooks/49

[CR26] Liu X, Wang H, Wang H, Guo Z, Xu X, Liu J, Wang S, Li W-X, Zou C, Prasanna BM, Olsen MS, Huang C, Xu Y (2018) Factors affecting genomic selection revealed by empirical evidence in maize. Crop J 6(4):341–352. 10.1016/j.cj.2018.03.005

[CR27] Lo S, Muñoz-Amatriaín M, Boukar O, Herniter I, Cisse N, Guo Y-N, Roberts PA, Xu S, Fatokun C, Close TJ (2018) Identification of QTL controlling domestication-related traits in cowpea (*Vigna unguiculata* L. Walp). Sci Rep 8(1):6261. 10.1038/s41598-018-24349-4 29674702 10.1038/s41598-018-24349-4 PMC5908840

[CR28] Lush JL (1943). Animal breeding plans. Animal Breeding Plans., Edn 2. https://www.cabdirect.org/cabdirect/abstract/19440100562

[CR29] Murphy RL, Klein RR, Morishige DT, Brady JA, Rooney WL, Miller FR, Dugas DV, Klein PE, Mullet JE (2011) Coincident light and clock regulation of pseudoresponse regulator protein 37 (PRR37) controls photoperiodic flowering in sorghum. Proc Natl Acad Sci U S A 108(39):16469–16474. 10.1073/pnas.1106212108 21930910 10.1073/pnas.1106212108 PMC3182727

[CR30] Nelimor C, Badu-Apraku B, Garcia-Oliveira AL, Tetteh A, Paterne A, N’guetta AS-P, Gedil M (2020) Genomic analysis of selected maize landraces from Sahel and Coastal West Africa reveals their variability and potential for genetic enhancement. Genes 11(9):1054. 10.3390/genes11091054 32906687 10.3390/genes11091054 PMC7565678

[CR31] R, H. A and B, M. F. J (1981). Quantitative genetics in maize breeding. 1st ed*.* https://agris.fao.org/search/en/providers/123819/records/64735c8a2c1d629bc97c8113

[CR32] Ramstein GP, Jensen SE, Buckler ES (2019) Breaking the curse of dimensionality to identify causal variants in breeding 4. Theor Appl Genet 132(3):559–567. 10.1007/s00122-018-3267-3 30547185 10.1007/s00122-018-3267-3 PMC6439136

[CR33] Ramstein GP, Larsson SJ, Cook JP, Edwards JW, Ersoz ES, Flint-Garcia S, Gardner CA, Holland JB, Lorenz AJ, McMullen MD, Millard MJ, Rocheford TR, Tuinstra MR, Bradbury PJ, Buckler ES, Romay MC (2020) Dominance effects and functional enrichments improve prediction of agronomic traits in hybrid maize. Genetics 215(1):215–230. 10.1534/genetics.120.303025 32152047 10.1534/genetics.120.303025 PMC7198274

[CR34] Rattunde F, Weltzien E, Sidibé M, Diallo A, Diallo B, Brocke K, Nebié B, Touré A, Traoré Y, Sidibé A, Diallo C, Diakité S, Bretaudeau A, Christinck A (2021) Transforming a traditional commons-based seed system through collaborative networks of farmer seed-cooperatives and public breeding programs: the case of sorghum in Mali. Agric Hum Values 38(2):561–578

[CR51] Rasmusson DC, Phillips RL (1997) Plant breeding progress and genetic diversity from de novo variation and elevated epistasis. Crop Science 37:303–310. 10.2135/cropsci1997.0011183X003700020001x

[CR35] Rutkoski JE (2019). A practical guide to genetic gain. In: Advances in Agronomy (Vol. 157, pp 217–249). Elsevier. 10.1016/bs.agron.2019.05.001

[CR53] Simmonds NW (1996) Family selection in plant breeding. Euphytica 90:201–208. 10.1007/BF00023859

[CR36] Swarup S, Cargill EJ, Crosby K, Flagel L, Kniskern J, Glenn KC (2021) Genetic diversity is indispensable for plant breeding to improve crops. Crop Sci 61(2):839–852. 10.1002/csc2.20377

[CR37] Technow F, Podlich D, Cooper M (2021) Back to the future: implications of genetic complexity for the structure of hybrid breeding programs. G3 Genes|genomes|genetics. 10.1093/g3journal/jkab153 10.1093/g3journal/jkab153 PMC8495936 33950172

[CR38] Tessele A, Morris GP, Akhunov E, Johnson BE, Clinesmith M, Fritz AK (2025) Interchromosomal linkage disequilibrium analysis reveals strong indications of sign epistasis in wheat breeding families. bioRxiv. 10.1101/2025.05.16.654524 10.1093/g3journal/jkaf265 PMC12774603 41206220

[CR39] Varshney RK, Shi C, Thudi M, Mariac C, Wallace J, Qi P, Zhang H, Zhao Y, Wang X, Rathore A, Srivastava RK, Chitikineni A, Fan G, Bajaj P, Punnuri S, Gupta SK, Wang H, Jiang Y, Couderc M, Xu X (2017) Pearl millet genome sequence provides a resource to improve agronomic traits in arid environments. Nat Biotechnol 35(10):969–976. 10.1038/nbt.3943 28922347 10.1038/nbt.3943 PMC6871012

[CR40] Viana JMS, Garcia AAF (2022) Significance of linkage disequilibrium and epistasis on genetic variances in noninbred and inbred populations. BMC Genomics 23(1):286. 10.1186/s12864-022-08335-9 35397494 10.1186/s12864-022-08335-9 PMC8994904

[CR41] Wade MJ (1992) Sewall Wright: gene interaction and the shifting balance theory. Oxf Surv Evol Biol 8:35–35

[CR42] Walker TS, Alwang J (2015). Crop improvement, adoption and impact of improved varieties in food crops in Sub-Saharan Africa. CABI. https://iaes.cgiar.org/spia/publications/crop-improvement-adoption-and-impact-improved-varieties-food-crops-sub-saharan

[CR43] Walsh B, Lynch M (2018a) Evolution and selection of quantitative traits. Oxford University Press, Oxford

[CR44] Walsh B, Lynch M (2018b) Family-based selection. In: Walsh B, Lynch M (eds) Evolution and selection of quantitative traits. Oxford University Press, Oxford

[CR45] Waples RK, Albrechtsen A, Moltke I (2019) Allele frequency-free inference of close familial relationships from genotypes or low-depth sequencing data. Mol Ecol 28(1):35–48. 10.1111/mec.14954 30462358 10.1111/mec.14954 PMC6850436

[CR46] Whiting JR, Paris JR, van der Zee MJ, Fraser BA (2022) AF-vapeR: a multivariate genome scan for detecting parallel evolution using allele frequency change vectors. Methods Ecol Evol 13(10):2167–2180. 10.1111/2041-210X.13952

[CR47] Wood TE, Burke JM, Rieseberg LH (2005) Parallel genotypic adaptation: when evolution repeats itself. Genetica 123(1–2):157–170 15881688 10.1007/s10709-003-2738-9 PMC2442917

[CR48] Wright S (1931) Evolution in mendelian populations. Genetics 16(2):97–159. 10.1093/genetics/16.2.97 17246615 10.1093/genetics/16.2.97 PMC1201091

[CR49] Wright, S. (1932). The roles of mutation, inbreeding, crossbreeding, and selection in evolution. Proc. 6th International Congress of Genetics, 1: 356–366.

[CR50] Yamaguchi M, Fujimoto H, Hirano K, Araki-Nakamura S, Ohmae-Shinohara K, Fujii A, Tsunashima M, Song XJ, Ito Y, Nagae R, Wu J, Mizuno H, Yonemaru J-I, Matsumoto T, Kitano H, Matsuoka M, Kasuga S, Sazuka T (2016) Sorghum Dw1, an agronomically important gene for lodging resistance, encodes a novel protein involved in cell proliferation. Sci Rep 6:28366. 10.1038/srep28366 27329702 10.1038/srep28366 PMC4916599

