# Supplementary material for: Genomic approaches to build de novo elite breeding gene pools from locally adapted landraces
Source: Theor Appl Genet. 2026 Jan 7;139(1):28. doi: 10.1007/s00122-025-05124-2 (PMC12774981; doi:10.1007/s00122-025-05124-2)
Supplement: Supplementary file 1 — Supplementary file1 (DOCX 18 KB) [file 122_2025_5124_MOESM1_ESM.docx]

## Table S1. Conceptual ambiguity in the definition of "eliteness*"* in representative contemporary and classical plant breeding literature

| **Authors** | **Year** | **Definition ^a^** |
| --- | --- | --- |
| Allier et al. ^1^ | 2020 | "Elite" not defined, but used 108 times in the text |
| Bernardo ^2^ | 2020 | Eliteness not discussed |
| Cobb et al. ^3^ | 2019 | "**Elite** **germplasm** can be defined as a reproductively compatible set of genotypes disproportionately enriched for favorable alleles that improve breeding value (i.e., the ***mean performance of the progeny*** of a given parent) in a particular environment or market." |
| Cobb et al. ^4^ | 2018 | "Elite" not defined, but used 61 times in the text |
| Lee ^5^ | 2015 | "**Elite** – a crop line that has ***many genes for good agronomic traits*** that result in high yields in a particular environment." |
| Falk ^6^ | 2010 | "Most breeding programs develop elite genotypes that are well adapted to the normal range of environmental conditions in the target production region. These **elite lines** have ***similar essential alleles*** for desirable end use characteristics, agronomics, disease resistance, and adaptation in the target region."  "The new lines with fewer defects are the elite lines that form the core of most breeding programs, and recombination among the progeny of elite elite crosses is the source of most new cultivars released by modern breeding programs" |
| Allard ^7^ | 1998 | "The main guiding principles for breeders ... can be summarized in three concepts. The *first* is that the most useful genetic resources in corn are the parents of modern **elite** single crosses that are ***well adapted*** in local environments. Natural selection, in combination with breeder-directed selection practiced over numerous generations, has increased the frequencies of favorable alleles in the parents of such single crosses and, more particularly, the frequencies of ***favorable epistatic combinations of alleles***, while decreasing the frequencies of less favorable alleles and the less favorable multiallelic combinations. But having many favorable alleles and favorable epistatic combinations of alleles present is not enough. Higher-order multilocus combinations of alleles, and particularly weblike combinations of alleles of different loci (Wright 1968) that tie much of the population genotype together, are also important. This leads to a *second* concept, namely, that once favorable multiallelic combinations have been developed for a given habitat, it is important that such ***combinations be preserved***, added to, and expanded to include additional favorably interacting loci. The most effective way to preserve favorable combinations is to hybridize **elite** materials with closely related **elite** relatives. As mentioned earlier, inbreeding or backcrossing in such hybrids is preferable to wider crosses, because the wider the cross, the greater the chances that segregation and recombination will dismantle previously developed favorable multilocus combinations. However, sooner or later the need will arise to introgress favorable alleles from nonrelated materials into such systems. The difficulties of doing this clearly depend on the numbers and the heritabilities of the alleles to be introgressed. The *third* concept is that discretely inherited marker alleles have increasingly provided breeders with effective means of identifying, tracking, and incorporating additional short regions of chromosomes with favorable effects into **elite** materials" (pg.151) |
| Poehlman & Sleper ^8^ | 1995 | Not defined in glossary or index. |
| Fehr ^9^ | 1991 | "The selection of parents for such characters generally involves selection of **elite germplasm** with the ***best performance for the character*** and the ***greatest genetic diversity available***" (pg. 122)  "Elite" not indexed, but appears 15 times in the text |
| Allard ^10^ | 1959 | Not defined in glossary or index. The "Choice of Parents" (pg. 116) refers to some concepts related to eliteness. |

^a^ A summary of explicit or implicit definitions on eliteness, and/or references to eliteness in each reference. Emphasis (bold and/or italics) is ours, not the authors.

^1^Allier, A., Teyssèdre, S., Lehermeier, C. *et al.* Optimized breeding strategies to harness genetic resources with different performance levels. *BMC Genomics* 21, 349 (2020). <https://doi.org/10.1186/s12864-020-6756-0>

^2^ Bernardo, R. Reinventing quantitative genetics for plant breeding: something old, something new, something borrowed, something BLUE. *Heredity* 125, 375–385 (2020). <https://doi.org/10.1038/s41437-020-0312-1>

^3^ Cobb JN, Juma RU, Biswas PS, Arbelaez JD, Rutkoski J, Atlin G, Hagen T, Quinn M, Ng EH. Enhancing the rate of genetic gain in public-sector plant breeding programs: lessons from the breeder's equation. Theor Appl Genet. 2019 Mar;132(3):627-645. doi: 10.1007/s00122-019-03317-0.

^4^ Cobb JN, Biswas PS, Platten JD (2018) Back to the future: revisiting MAS as a tool for modern plant breeding. *Theor Appl Genet.* https://doi.org/10.1007/s00122-018-3266-4

^5^ Lee, M. (2015). Plant breeding and crop improvement.

^6^ Falk, D. E. (2010). Generating and maintaining diversity at the elite level in crop breeding. *Genome*. 53(11): 982-991. <https://doi.org/10.1139/G10-081>

^7^ Allard, R. W. (1998). *Principles of Plant Breeding*. New York: John Wiley & Sons. (2nd Edition)

^8^ Poehlman, J. M., & Sleper, D. A. (1995). *Breeding Field Crops* (4th ed.). Ames, Iowa: Iowa State University Press.

^9^ Fehr, W. R. (1991). *Principles of Cultivar Development: Theory and Technique* (Vol. 1). Ames, Iowa: Iowa State University Press.

^10^ Allard, R. W. (1959). *Principles of Plant Breeding*. New York: John Wiley & Sons. (1st Edition)

#

#

# Table S2. Estimated field phenotyping budget per cycle for 404 pearl millet lines from the national core collection at the ISRA research station in Bambey, Senegal.

|  | **Activities included** | **Cost (FCFA)** | **Cost (USD)** |
| --- | --- | --- | --- |
| 1. Field preparation | Plowing | 25,000 | $42 |
| 1. Inputs | Fertilizers, pesticides, and others products | 150,000 | $250 |
| 1. Labor | Temporary labor, Daily labor | 3,200,000 | $5,333 |
| 1. Material and supplies | Small material, Bags, Labels | 200,000 | $332 |
| Total |  | 3,525,000 | $5,875 |
| **Total per line** |  | **38,315** | **$63** |
| **Total per plant** |  | **8,725** | **$22** |

#

# Costs are presented in both the original currency (CFA Franc) and in US dollars, assuming a conversion rate of 1 USD = 600 FCFA.

## Table S3. Estimated per line costs of phenotypic and genotypic screening strategies for characterizing de novo elite gene pools in a nascent breeding program

| **Analysis ^a^** | **Cost (USD/line)** |
| --- | --- |
| FPI | $904 |
| QGI, assuming 4 known QTL | $85 |
| QGI with linkage mapping to identify QTL | $670 |
| QGI with GWAS to identify QTL | $843 |
| PGI | $125 |

^a^ FPI (Family-based Phenotypic Inference): Relies on replicated phenotyping across environments; cost is per line per location.

## Table S4. Estimated screening costs for QTL-based genotypic inference (QGI) across varying numbers of known QTL

| **Analysis ^a^** | **Cost (USD) ^b^** |
| --- | --- |
| QGI_2 | $75 |
| QGI_4 | $85 |
| QGI_20 | $165 |

^a^ Numbers indicate QTL count per scenario.

^b^ Costs assume prior knowledge of QTL and are based on the use of KASP markers for genotyping.
